# Exciton polariton interactions in Van der Waals superlattices at room temperature

**DOI:** 10.1038/s41467-023-36912-3

**Published:** 2023-03-17

**Authors:** Jiaxin Zhao, Antonio Fieramosca, Kevin Dini, Ruiqi Bao, Wei Du, Rui Su, Yuan Luo, Weijie Zhao, Daniele Sanvitto, Timothy C. H. Liew, Qihua Xiong

**Affiliations:** 1 Division of Physics and Applied Physics, School of Physical and Mathematical Sciences, Nanyang Technological University, Singapore, 637371 Singapore; 2 State Key Laboratory of Low-Dimensional Quantum Physics and Department of Physics, Tsinghua University, 100084 Beijing, P.R. China; 3 School of Physics, Frontiers Science Center for Mobile Information Communication and Security, Southeast University, 211189 Nanjing, P.R. China; 4 Purple Mountain Laboratories, 211111 Nanjing, P.R. China; 5 CNR NANOTEC Institute of Nanotechnology, via Monteroni, Lecce, 73100 Italy; 6 INFN National Institute of Nuclear Physics, Lecce, 73100 Italy; 7 MajuLab, International Joint Research Unit UMI 3654, CNRS, Université Côte d’Azur, Sorbonne Université, National University of Singapore, Nanyang Technological University, Singapore, Singapore; 8 Frontier Science Center for Quantum Information, 100084 Beijing, P.R. China; 9 Beijing Academy of Quantum Information Sciences, 100193 Beijing, P.R. China; 10 Collaborative Innovation Center of Quantum Matter, 100084 Beijing, P.R. China

**Keywords:** Polaritons, Two-dimensional materials

## Abstract

Monolayer transition-metal dichalcogenide (TMD) materials have attracted a great attention because of their unique properties and promising applications in integrated optoelectronic devices. Being layered materials, they can be stacked vertically to fabricate artificial van der Waals lattices, which offer unique opportunities to tailor the electronic and optical properties. The integration of TMD heterostructures in planar microcavities working in strong coupling regime is particularly important to control the light-matter interactions and form robust polaritons, highly sought for room temperature applications. Here, we demonstrate the systematic control of the coupling-strength by embedding multiple WS_2_ monolayers in a planar microcavity. The vacuum Rabi splitting is enhanced from 36 meV for one monolayer up to 72 meV for the four-monolayer microcavity. In addition, carrying out time-resolved pump-probe experiments at room temperature we demonstrate the nature of polariton interactions which are dominated by phase space filling effects. Furthermore, we also observe the presence of long-living dark excitations in the multiple monolayer superlattices. Our results pave the way for the realization of polaritonic devices based on planar microcavities embedding multiple monolayers and could potentially lead the way for future devices towards the exploitation of interaction-driven phenomena at room temperature.

## Abstract (web summary)

The authors embed a multiple quantum-well WS_2_ heterostructure in a planar microcavity and show the systematic control of the normal mode coupling-strength. They find a strong enhancement of the characteristic time scale, which they attribute to long-lived dark excitations emerging in the structure.

## Introduction

Monolayer (ML) group-VI transition-metal dichalcogenides (TMDs), owing to their exceptionally large exciton binding energies as well as valley degrees of freedom, have shown great potential for a wide range of valleytronic and optoelectronic applications^1,2^. TMDs can be exfoliated from the bulk crystal down to the ML level and subsequently stacked together, thus forming heterostructures in which the weak van-der-Waals forces are sufficient to keep the layers packed. The fabrication of TMD-heterostructures has become an exciting route for the realization of artificial materials with tailorable electronic and optical properties. For instance, multiple TMD-ML separated by hexagonal Boron Nitride (h-BN) flakes have been used to fabricate a coupled quantum-well structure for tunneling diodes while the formation of interlayer excitons and trions has been evidenced by stacking two different TMDs^3^. Moreover, by controlling the crystallographic alignment between the two crystals, Moirè excitons have also been reported^4–8^.

Typically, these kinds of heterostructures are exploited to realize excitonic devices for photonics and opto-electronics application^9,10^, but great interest is growing towards the integration in microcavities, thereby offering new possibilities to control the light–matter interactions. In this respect, the study of the strong-coupling regime, i.e., the formation of hybrid light–matter quasiparticles called exciton-polaritons^11,12^, is particularly attractive. Owing to their low effective mass and strong nonlinearity, polaritons have been employed in the study of Bose–Einstein condensates^13,14^ and superfluidity^15,16^ as well as in proof-of-concept photonic and optoelectronic devices^17^. Their properties open up new and exciting perspectives towards the realization of ultralow-power all-optical polariton devices and because of the robust excitonic transition, TMDs are excellent candidates to bring the polariton physics at room temperature^18–25^. Compared with other materials on the exciting frontier of room-temperature polaritonics^22,26–33^, TMD microcavities offer exceptional stability under optical excitation besides being readily tunable via external electric and magnetic fields.

The integration of van der Waals superlattices in planar microcavity could enable the realization of polaritonic devices with remarkable functionalities, but nonetheless only a few examples reported the integration of complex heterostructure in planar microcavities. For example, a double-layer heterostructure (two MoSe_2_-MLs) has been used in an open cavity system to study the strong coupling^34^; three layers have been integrated into a polariton light-emitting diode (three WS_2_-MLs)^20^ as well as to observe the strong-coupling of Rydberg exciton states (three WSe_2_-MLs)^35^. Moreover, by controlling the crystallographic alignment of a WS_2_/MoSe_2_ stack, the formation of Moirè polaritons has been recently observed^36^.

The study of nonlinear optical properties, which is still not completely understood in TMD-based microcavities at room temperature, is crucial for the development of fascinating polariton physics and controllable polaritonic devices. New insights in this direction can be obtained by employing time-resolved pump-probe techniques, which are capable of discerning interparticle Coulomb interaction from phase space filling (PSF) effects. Moreover, time-resolved pump-probe techniques are also powerful tools to reveal the presence of dark excitations, which contribute to the energy renormalization of the polariton dispersion in the nonlinear regime, therefore providing new critical information about the many-body physics in TMD polaritons. For instance, the presence of dark states uncoupled from light can be observed in planar microcavities embedding multiple MLs^37^, and their long-living nature could be exploited for the realization of polaritonic circuits^38^, thus overcoming the limitation imposed by the short polariton lifetime of the state-of-the-art TMD microcavities. Therefore, systematic control of the number of stacked layers is important to explore this route, which not only offers an additional degree of freedom on the tunability of the coupling strength^39^, but also provides new possibilities to exploit stable polaritons towards the realization of TMD-based devices.

Here, we report the observation of strong coupling in microcavities embedding up to four WS_2_-MLs separated by an insulating layer. The observed Rabi splitting exhibits an enhancement proportional to the square root of the number of stacked MLs and reaches ~72 meV for the four-fold structure. We studied the nonlinear properties of the samples by pumping the ground state and probing the polariton dispersion around the crossing point, revealing that a reduction of the oscillator strength is the main mechanism behind polariton interaction at room temperature. We have also estimated the value of the interaction and saturation constants. Moreover, through the use of time-resolved pump-probe measurements, we observed the presence of long-living dark states in the multiple MLs sample which lasts up to 30 times longer than the bright polaritons and substantially contributes to the energy renormalization in the nonlinear regime. These dark states arise, as their bright counterpart, from the linear superposition of the ML bright excitons. While the bright multilayer states are in-phase superpositions, the dark ones, populated through the scattering of the bright multilayer excitons, are out of phase leading to decoupling to light and therefore have a longer lifetime. These multilayer dark states are different from the intralayer or interlayer dark exciton states, which have completely different behavior and properties.

## Results

### Optical characterization and fabrication of microcavity

**Fig. 1:**
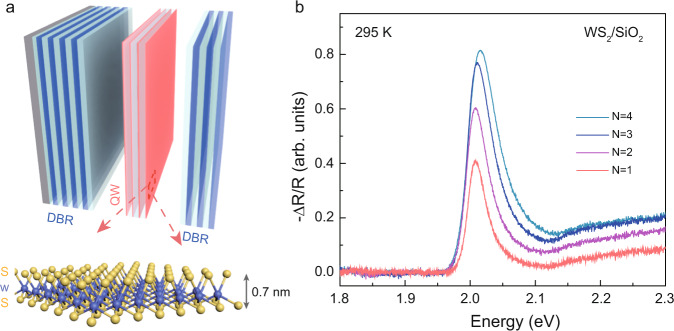
Schematic of the multiple MLs microcavity and optical characterization of the bare heterostructure. **a** Schematic image of the full dielectric microcavity structure embedding multiple WS_2_-MLs. **b** Reflectance contrast (−Δ*R*/*R*) of the bare superlattices with a different number of stacked MLs before closing the microcavity.

The sample structure consists of a WS_2_-heterostructure embedded in a full-dielectric planar microcavity made of two distributed Bragg reflector (DBR) mirrors and two SiO_2_ spacers, as schematically shown in Fig. 1a. The DBRs are fabricated by alternating titanium dioxide (TiO_2_) and silicon dioxide (SiO_2_) layers (10.5 pairs) grown on top of a silicon wafer. The WS_2_-MLs are mechanically exfoliated from the bulk crystal and subsequently assembled into heterostructures with a different number of MLs directly on top of the bottom mirror. From now on we refer to the heterostructure as superlattice-N2, -N3, and -N4. A SiO_2_ layer or hBN is used as an insulator (Supplementary Figs. 1 and 2). In both cases, the thickness of the insulating layer is chosen to avoid electronic coupling and tunneling between the different TMD-MLs and is ~6 nm for SiO_2_ while it varies from 5 up to 7 nm for hBN^40^. Further fabrication details are presented in the “Methods” section. The superlattice is placed in the center of the planar microcavity, i.e., the maximum intensity of the electromagnetic field, while the energy of the cavity mode is tuned by controlling the thickness of the two SiO_2_ spacers. Before closing the microcavity, reflectivity measurements were carried out to determine the optical quality of the superlattices. An increase in the oscillator strength is visible by increasing the number of MLs, as shown by the differential reflectivity spectra, $\varDelta R/R$, reported in Fig. 1b. The photoluminescence spectra evidence the presence of neutral excitons at 2.006 eV with an inhomogeneous broadened full-width half-maximum (FWHM) of ~38 meV, and trions at 1.975 eV with an FWHM of ~64 meV, as reported in Supplementary Fig. 3 for all the fabricated superlattices. In all the investigated samples the quality factor of the bare cavity mode is ~1000 (Supplementary Fig. 4).

### Strong coupling

**Fig. 2:**
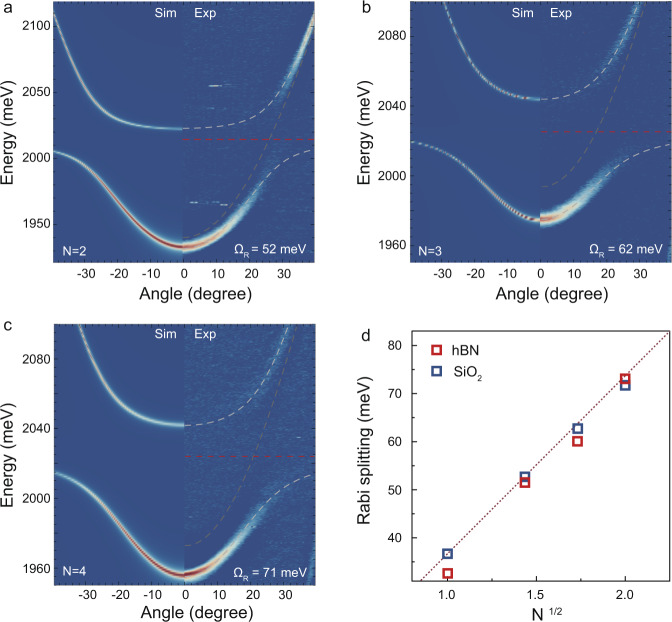
Exciton-polaritons formation in superlattice microcavity. Angle-resolved reflectivity maps for the superlattice-N2 (**a**), -N3 (**b**), and -N4 (**c**), respectively (with SiO_2_ insulating spacer). The left (right) part of the figures shows the TMM calculations (experiments). The two dashed white curves are the UPB and LPB dispersions obtained by using a coupled oscillator model. The dashed red line and dashed gray parabola represent the exciton energy and the dispersion of cavity mode, respectively. **d** Rabi splitting as a function of the square root of the number of layers for different insulators, hBN (red) and SiO_2_ (blue). A clear $\sqrt{{N}_{{{\rm {layer}}}}}$ enhancement of the coupling strength is found.

We carried out angle-resolved reflectivity measurements along with transfer matrix simulations (TMM) to confirm the presence of the strong-coupling regime, as shown in Fig. 2a–c, for the superlattice-N2, -N3, and -N4 made with SiO_2_ spacer, respectively. The reflectivity map of the ML sample is reported in previous work^22^, while the reflectivity of the superlattices made with an hBN spacer is shown in Supplementary Fig. 5. The dispersion of the cavity mode (gray dashed parabola) is bending around the excitonic resonance (red dashed line), highlighting the presence of two new branches, i.e., the upper (UPB) and lower (LPB) polariton branch (white dashed lines), therefore confirming the formation of polaritons in superlattice microcavities. We used a coupled oscillator model to fit the data and evaluate the vacuum Rabi splitting (${\varOmega }_{{\rm {R}}}$), that is, ~52, ~62, and ~72 meV for the superlattice-N2, -N3, and -N4, respectively. In strongly coupled systems embedding multiple layers (such as GaAs and CdTe multiple quantum-wells microcavities) the ${\varOmega }_{{\rm {R}}}$ is well approximated by the following expression^41^: ${\varOmega }_{{\rm {R}}} \propto \sqrt{\frac{{f}_{{{\rm {osc}}}}{N}_{{{\rm {layer}}}}}{{L}_{{{\rm {eff}}}}}}$, where ${f}_{{{\rm {osc}}}}$ is the oscillator strength of the active material and ${L}_{{{\rm {eff}}}}$ is the optical length of the cavity. A square root dependance is expected for the Rabi splitting as a function of the number of embedded layers ${N}_{{{\rm {layer}}}}$. Figure 2d summarizes our experimental observations, which perfectly follow the expected trend for both insulating materials. It is important to note that the insulating spacer is fundamental to preserve the resonance width, avoid a decrease in the oscillator strength as well as maintain the direct-bandgap electronic structure. Indeed, a deviation from the square root dependance has been observed in multilayer samples without a spacer, which exhibits an indirect bandgap^42^. Our results clearly show that a precise and tailored control of the light–matter interactions is  achieved.

### Nonlinearity

**Fig. 3:**
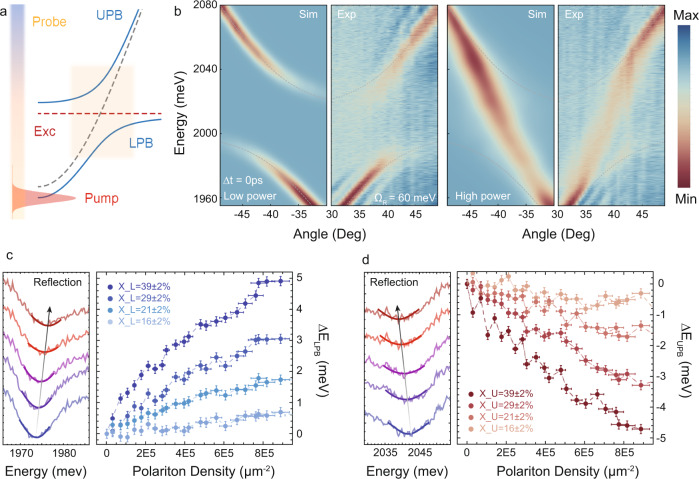
Polariton nonlinear interaction in the superlattice-N3. **a** Scheme of the pump-probe experiment in which the pump injects polaritons into the ground state and the probe (broad in energy) monitors the UPB and LPB dispersion at high momenta. **b** Angle-resolved reflectivity maps for low and high pumping power, respectively. The left (right) part of the figures shows the calculations (experiments). Power sweep of the LPB (**c**) and UPB (**d**)  reflectivity energy profiles extracted at the same excitonic fraction arbitrarily shifted along the vertical axis. Energy renormalization of the LPB (**c**) and UPB (**d**) as a function of the pumping fluence for different excitonic fractions. The error bars of polariton density in **c** and **d** are explained in the “Methods” section. The error bars on the energy shift data correspond to the 95% confidence interval of the gaussian fit.

We then focused the attention on the nonlinear properties. In the polariton framework, there are two primary mechanisms that play a central role in polariton nonlinearities: interparticle Coulomb interactions and phase space filling (PSF). The first one, characteristic of III–V semiconductors, leads to a blueshift of the exciton resonance and consequently to a blueshift of both UPB and LPB. The second, typical of organic systems, leads to a reduction of the oscillator strength which is reflected in a blueshift of the LPB and a redshift of the UPB. Therefore, to reveal the main mechanism behind polariton nonlinearities, it is mandatory to study the energy shift of both branches. We used a pump-probe technique in which the pump (~100 fs, 1 kHz) injects polaritons into the ground state, while a broadband probe is used to monitor the energy shift of the two branches at high momenta, as schematically shown in Fig. 3a. Significantly, our excitation scheme minimizes the direct formation of the excitonic reservoir and ensures to deal mainly with polariton states^19,29^. Although, we found the same Rabi splitting scaling law for both SiO_2_ and hBN spacers, for the study of nonlinearities we only used the sample made with hBN. This choice is dictated by the better visibility at high in-plane momenta offered by the hBN encapsulation, which is crucial in our experiment. The lower visibility of the SiO_2_ sample can be explained in terms of higher disorder, and therefore higher optical losses, compared to the hBN spacer.

The two beams are overlapped in space in a spot with a diameter of ~3 μm and matched in time by using a motorized delay stage. A detailed scheme of the optical setup is reported in Supplementary Fig. 6. The angle-resolved reflectivity maps of the superlattice-N3, collected at $\varDelta t=0\,{{\mbox{ps}}}$ and low (high) pumping power, are shown in Fig. 3b (3c). As it is clear from the high-power map, a complete quenching of the Rabi splitting is eventually obtained at high pumping power, which immediately suggests the dominant role of saturation in TMD-based heterostructures. The theoretical calculation is shown in the left part of the maps, in good agreement with the experiments. The model we consider consists of the description of three separated 2D ML excitons modes ${\chi }_{j}(j={{{{\mathrm{1,2,3}}}}})$ and the single optical cavity mode ${\varPsi }_{\gamma }$, through renormalized non-linear coupled Schrodinger equations:

1 $$i{{\hslash }}\frac{\partial {\psi }_{\gamma }}{\partial t}=\left(-\frac{{{{\hslash }}}^{2}{\nabla }^{2}}{2{m}_{\gamma }}+\varDelta -i\gamma \right){\psi }_{\gamma }+\mathop {\sum } \limits _{n=1}^{N}\left(\frac{\varOmega }{2}-\frac{\rho \alpha }{2}{\left|{\chi }_{n}\right|}^{2}\right){\chi }_{n}+P+{\eta }_{\gamma }$$

2 $$i{{\hslash }}\frac{\partial {\chi }_{j}}{\partial t}=\frac{\varOmega }{2}{\psi }_{\gamma }+\alpha {\left|{\chi }_{j}\right|}^{2}{\chi }_{j}-\frac{\rho \alpha }{2}{\chi }_{j}^{2}{\psi }_{\gamma }^{*}-\rho \alpha {\left|{\chi }_{j}\right|}^{2}{\psi }_{\gamma }+{\eta }_{j}+{\mu }_{j}{{{{{{\rm{\chi }}}}}}}_{{{{{{\rm{j}}}}}}}$$

where ${m}_{\gamma }$is the effective mass of the cavity photon; Δ is the exciton-photon detuning; $\gamma$ is the photon decay rate; $\varOmega$ is the Rabi splitting, $\alpha$is the polariton–polariton interaction, and $\rho$ is the ratio between the saturation and the interaction constants. This ratio is directly evaluated from the experimental data (as discussed below) and is of the same order as the one obtained in recently published estimations^42^. The parameters of the local pulsed resonant pump *P* are taken from the experimental data. The complex noises *η* are different for each mode and play the role of the broadband weak probe in the simulations. The random disorder potentials ${\mu }_{j}$ are then taken as fitting parameters as they are responsible for the scattering to the dark states. Time-evolving these equations from small intensity random initial conditions and averaging over 1000 different realizations for the noise and the disorder, we obtain the dynamics of the polaritons over 50 ps. We then Fourier transform in time and space the signal to obtain the dispersion. The other parameters are found referring to the TMM simulations. Once the right parameters were found for the low-intensity calculations, we tuned the pump amplitude to fit the results obtained by the experiments.

The quenching of the Rabi splitting is better highlighted in Supplementary Movie 1 as well as in the energy profiles reported in the left part of Fig. 3c, d for the LPB and UPB, respectively, which show the full power sweep. The energy shift of the two branches as a function of the pumping power and different excitonic fractions is reported in the right part of Fig. 3c, d. The LPB (blue points, Fig. 3c) blueshifts while the UPB (red points, Fig. 3d) clearly redshifts. The two branches show a perfect specular trend where the magnitude of the energy shift increases by increasing the excitonic fraction, thus suggesting that PSF nonlinearity is the primary mechanism of interaction in the WS_2_ superlattice-N3. We note that two recent works suggested that saturation contributes significantly to polariton nonlinearities in a MoSe_2_ ML microcavity at low temperature as well as a plasmonic cavity^43,44^. Moreover, the contribution from Coulomb interaction has been seen only at a cryogenic temperature so far^36^. Our results are in good agreement with these observations and clearly demonstrate that the dominant mechanism behind polariton nonlinearities in planar microcavities embedding multiple MLs at room temperature arises from PSF nonlinearity. The same experiment has been carried out for the ML microcavity, as shown in Supplementary Fig. 7 and Supplementary Movie 2. From the data reported in Supplementary Fig. 7, Fig. 3c, d we can estimate the value of the exciton–exciton interaction and saturation constants for the ML and superlattice-N3, thus retrieving the above-reported ratio, *ρ*, between the two constants. In particular, we have first estimated the polariton density in the LPB (see the “Methods” section) and then, by following the procedure already developed in the past^42^, we expressed the energy shift of the two branches depending on the exciton fraction and the polariton population, assuming negligeable population in the UPB:

3 $${\varDelta }_{{\rm {L}}}={X}_{{\rm {L}}}^{2}n{g}_{{{\rm {exc}}}}+{X}_{{\rm {L}}}^{2}{({X}_{{\rm {L}}}{C}_{{\rm {L}}})}^{1/2}n{g}_{{{\rm {sat}}}}$$

4 $${\varDelta }_{{\rm {U}}}=2{X}_{{\rm {U}}}{C}_{{\rm {U}}}n{g}_{{{\rm {exc}}}}-{C}_{{\rm {U}}}^{2}{({X}_{{\rm {U}}}{C}_{{\rm {U}}})}^{1/2}n{g}_{{\rm {s{at}}}}$$

where ${\varDelta }_{{\rm {L/U}}}$ is the energy shift of the LPB/UPB, ${X}_{{\rm {L/U}}},{C}_{{\rm {L/U}}}$ are, respectively, the excitonic and photonic fraction for the LPB and UPB at the point of measure, calculated by using the coupled oscillator model. *n* is the population in the LPB distributed over the number of stacked layers. From these expressions, we extract the value of the interaction constant ${g}_{{{\rm {exc}}}}$ and the saturation constant ${g}_{{{\rm {sat}}}}$. The values are averaged over different excitonic fractions and pumping powers while the errors represent the maximum/minimum deviation from the average value.

The extracted constants for the ML sample are ${g}_{{{\rm {exc}}}} \sim 0.055\pm 0.015\,\mu {{\rm {eV}}}\,\cdot\mu {{\rm {m}}}^{2}$ and ${g}_{{{\rm {sat}}}} \sim 0.11\pm 0.035\,\mu {{\rm {eV}}}\cdot \mu {{\rm {m}}}^{2}$, in good agreement with previous reports^19,45^, while the superlattice-N3 shows a weaker interaction constant which is ${g}_{{{\rm {exc}}}} \sim 0.01\pm 0.008\,\mu {{\rm {eV}}}\cdot \mu {{\rm {m}}}^{2}$ and ${g}_{{{\rm {sat}}}} \sim 0.04\pm 0.01\,\mu {{\rm {eV}}}\cdot \mu {{\rm {m}}}^{2}$. Our experimental estimations are in good agreement with a recent theoretical proposal which suggested a weaker interaction constant for TMD superlattices with multiple stacked MLs^37^. This can be explained considering the spin-triplet (same spin) and spin-singlet (opposite spin) interaction strengths present in TMD-superlattices for the contact interaction term. In particular, the increase of the number (*N*) of stacked layers suppresses the polariton triplet interaction strength (suppression proportional to ∼*N*) thus lowering the interaction constant. Moreover, although ref. ^37^ only describes the reduction of the contact interaction term, our experimental data also show a reduction of the saturation term for the superlattice sample. This is expected, as samples with multiple monolayers contain more exciton states. Therefore, the required area density to fill them all is higher, which turns in a lower space-filling nonlinearity compared to the single monolayer microcavity. The modification of the spin-triplet and spin-singlet interaction strength by changing the number of stacked layers provides an additional degree of freedom for the realization of interaction-driven phenomena, such as polariton blockade and superfluidity as well as the study of spin-polarized domains, which are intrinsically related to the magnitude and sign of these kinds of interactions.

### Temporal dynamics

**Fig. 4:**
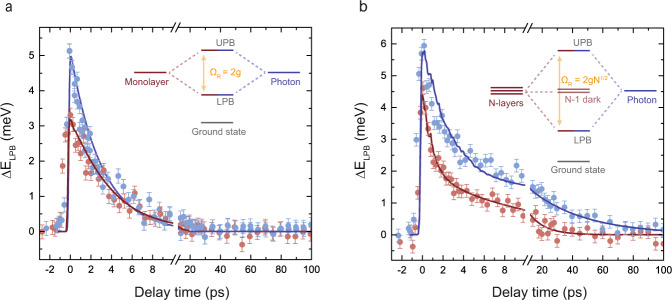
Comparison of the temporal dynamics for the ML microcavity and superlattice-N3. **a** LPB blueshift as a function of the time delay for low (red) and high (blue) pumping power. The dots represent the experimental points while the solid lines are the fit considering the model reported in the main text, which shows a single exponential decay. Inset: schematic illustration of the polariton eigenstates in the single ML microcavity, which consist of two bright polariton modes (UPB and LPB) separated by the Rabi energy, Ω_R_ = 2 g. **b** Same as in **a** but for the superlattice-N3. The LPB blueshift as a function of the time delay can be well approximated by a two-exponential decay. The polariton eigenstates in the superlattice-N3 microcavity consist of two bright polariton modes (UPB and LPB) separated by the Rabi energy, Ω_R_ = 2 gN^1/2^ along with N-1 uncoupled dark states located at the exciton energy. The error bars on the energy shift data correspond to the 95% confidence interval of the gaussian fit.

Compared with the well-developed GaAs-based polariton microcavities, one of the main drawbacks of TMDs-polaritons is represented by the short polariton lifetime^22,46^. The dark excitations, which usually exhibit a long lifetime, could represent an interesting strategy to overcome this limitation, thus facilitating the realization of polariton devices. However, in contrast to the common optical spectroscopy, which allows to gain information only about the bright transitions, revealing the presence of dark excitations is in general more complicated. Here, by taking advantage of the pump-probe configuration, we investigated the temporal dynamics of the nonlinearities, which are sensitive to the presence of such excitations. Indeed, the dark states contribute to the interactions and thus can be detected by monitoring the energy renormalization of the probe spectrum in time. It is important to note that here we are referring to dark excitations that only exist in the multilayer structures. In particular, when several MLs (*N*) are taken into account, the eigenstates of the system consist of two bright polariton modes (UPB and LPB) and *N*−1 dark states which are out of phase and decoupled from light^47,48^. Their energy lies at the exciton energy and, although treated as virtual states in the theory, they can be turned into real long-lived excitations via phonon-assisted scattering. The energy level scheme for the ML sample and superlattice-N3 are reported in the insets of Fig. 4a, b, respectively.

Therefore, we compared the behavior of the ML and superlattice-N3 by tracking the energy shift of the LPB, $\varDelta E{\left(t,P\right)}_{{{\rm {LPB}}}}$, as a function of the time delay between the pump and probe. The ML sample (*X* ~ 0.4) exhibits a sharp increase of the blueshift (expected for an fs pulsed excitation), which decreases exponentially at long delay times, as reported in Fig. 4a for low (red points) and high (blue points) pumping power. The kinetics of the blueshift can be well approximated considering only the presence of bright excitons, i.e., a single-exponential decay, in the above-reported model, as shown by the red and blue solid lines in Fig. 4a. Therefore, we retrieve a decay time of $t=3.8\pm 0{{\mbox{.}}}2\,{{\mbox{ps}}}$ and $t=3.2\pm 0{{\mbox{.}}}2\,{{\mbox{ps}}}$ for low and high pumping power, respectively. We note here that the observed single exponential decay it is in good agreement with previous reports^44,49^. Moreover, similar behavior is also confirmed by using different monolayer microcavities as shown in Supplementary Figs. 8 and 9. In contrast, the superlattice-N3 (*X* ~ 0.4), although showing a similar fast blueshift rise time, exhibits a complex temporal dynamic, as reported in Fig. 4b for low (red dots) and high (blue dots) pumping power, respectively. In this case, in order to capture the complex decay behavior of $\varDelta E{\left(t,P\right)}_{{{\rm {LPB}}}}$ in the above-reported model, we have taken into account the presence of dark excitations arising from the scattering of the bright excitons into dark states that are out of phase and decoupled with light. These dark states cannot decay radiatively and decay mostly through electron–hole dissociation or coupling back to bright states. The dark state decay time is taken to be longer than the bright state decay time (with little sensitivity on the exact value, as shown in Supplementary Fig. 10). Then, we can well approximate the experimental data obtaining biexponential kinetics with a fast (*t*_1_) and a slow (*t*_2_) component. Specifically, we obtain $t_1=2.5\pm 0{{\mbox{.}}}5\,{{\mbox{ps}}}$ and $t_2=22\pm 5\,{{\mbox{ps}}}$ at low pumping power, while the values are $t_1=3\pm 0{{\mbox{.}}}5\,{{\mbox{ps}}}$ and $t_2=57\pm 7\,{{\mbox{ps}}}$ at high pumping, respectively. To underline the differences between the exponential vs. bi-exponential fitting in the same scale, as shown in Supplementary Fig. 11, we plot the blueshift as a function of delay time without any break in the *x*-scale. Moreover, we also observed a clear dependance of the dark state decay time as a function of the excitonic fraction, as shown in Supplementary Fig. 12. In particular, the slow decay time ranges from $30\pm 5\,{{\mbox{ps}}}$ at low excitonic fraction (*X* ~ 0.2) up to $80\pm 5\,{{\mbox{ps}}}$ at high excitonic fraction (*X* ~ 0.5), while the fast decay component is not strongly affected. This is consistent with the above-drawn picture, where a higher scattering rate between bright and dark polaritons is expected by increasing the excitonic fraction, thus populating the dark states more intensively.

It is important to note that the above-presented differences in the decay time between the ML and the superlattice-N3 strongly point toward the observation of dark states sustained by the superlattices. Indeed, although we cannot completely rule out the presence of long-living excitonic complexes, these species would be formed regardless of the number of stacked layers, thus giving rise to a long decay time even in the ML sample, which instead is not the case of Fig. 4a. Moreover, we further stress that the pump laser injects polaritons into the ground state (far in energy and redshifted with respect to the exciton resonance), thus minimizing the formation of parasitic exciton complexes that would affect the temporal blueshift dynamics.

Our experimental observations and theoretical calculations confirm the presence of long-living dark excitations in TMDs heterostructure with multiple layers, with a decay time longer than the brighter counterpart. The observation of such a longer lifetime makes them an interesting possibility to imprint spatial potentials in the microcavity plane that can be exploited in polariton circuits. It is important to note that the blueshift decay time could be further enhanced by stacking several monolayers as well as by controlling the amplitude of the disorder, as reported in Supplementary Fig. 13, therefore leading to the enhancement necessary for realistic applications. In this respect, the use of chemical vapor deposition or molecular beam epitaxy technique would facilitate the growing of several layers and the introduction of layers with controlled roughness, which would make it possible to enhance and carefully control the blueshift-induced lifetime. However, the increasing disorder may lead to a broadening of the polariton mode and eventually, an optimum disorder level is required to get a trade-off between the blueshift-induced lifetime and the broadening of the polariton mode.

## Discussion

In summary, we have demonstrated the systematic control of the coupling strength in polariton microcavities embedding TMD-superlattices with multiple MLs (up to four). The observed Rabi splitting perfectly scales as the square root of the number of layers and is enhanced up to 72 meV in the four-fold microcavity, making the cavity polaritons more stable. By studying the interaction properties, we have shown that PSF is the main mechanism behind polariton interaction and that the saturation constant is higher than the exciton–exciton interaction in TMD-superlattices at room temperature. Moreover, by using a time-resolved pump-probe technique we revealed the presence of long-living dark states arising from the scattering of bright excitons into dark states, which are out of phase and decoupled with light. In contrast to single ML microcavities, a decay time of up to 30 times longer has been found in superlattices with multiple MLs. Our results shed light on the comprehension of the many-body physics of polariton TMDs microcavities and could potentially lead the way for the realization of interaction-driven phenomena at room temperature. Moreover, we anticipate here that the long lifetime observed in multilayer devices can be exploited for the realization of polariton circuits in which the dark states could be used to imprint spatial potentials, thus opening an interesting route for future research and applications.

## Methods

### Experimental structure

The WS_2_ superlattice microcavity consists of a ${\lambda }_{{{\rm {Exc}}}}/2$ cavity sandwiched between two DBRs. The bottom DBR mirror is composed of 10.5 alternating pairs of silicon dioxide (SiO_2_) and titanium dioxide (TiO_2_) deposited on a silicon wafer by using e-beam evaporation (Cello, Ohmiker-50B). Then, the first ${\lambda }_{{{\rm {Exc}}}}/4$-SiO_2_ spacer was deposited onto the first DBR by using the same technique. The multiple ML structure is made by using several WS_2_ MLs separated by a thin SiO_2_ layer with a thickness of around ~6 nm. The ML was mechanically exfoliated from the commercial bulk WS_2_ crystals (HQ Graphene). For details, the first ML was isolated and transferred onto the bottom DBR through a dry polymer transfer process. Then, the thin SiO_2_ layer was deposited via e-beam evaporation. Next, the second ML was exfoliated on a PDMS layer (PF-X4, Gel-Pack). The top WS_2_/PDMS film was aligned with the first ML by using a home-built transfer stage and placed onto the surface. After removing the PDMS layer, the second ML was left on top of the SiO_2_ layer and aligned with the first ML. As for the hBN encapsulated samples, the ML WS_2_ and few-layer hBN flakes (HQ Graphene) are prepared by mechanical exfoliation from bulk crystals. Heterostructure stacking and transfer are done by using the polypropylene carbonate transfer technique^50^. The superlattice structure was realized by following this process for several times. Finally, the second ${\lambda }_{{{\rm {Exc}}}}/4$-SiO_2_ spacer and the top DBR with 6.5 pairs were deposited by using the e-beam evaporation.

### Optical characterization

The angle-resolved reflectivity maps shown in Fig. 2, were measured in a home-built setup in the Fourier imaging configuration by using a broadband white light source. A high numerical aperture microscope objective (NA = 0.9, 100X) was used. The reflected light from the microcavity was collected through the narrow slit of the spectrometer (Horiba, iHR550), equipped with a grating (600 lines/mm) and coupled to a 2D charge-coupled device (CCD) array (Horiba, Symphony II). The measurements shown in Figs. 3 and 4 were conducted by employing a home-built pump-probe setup (Supplementary Fig. 6). The 800 nm output pulsed laser (1 kHz repetition rate, ~100 fs pulse width) from a Ti: Sapphire regenerative amplifier was split into two paths. One beam went through a mechanical delay stage and pumps a sapphire crystal to generate a broadband light. The broadband light served to probe the energy shift of the UPB and LPB. The second beam was sent to an optical parametric amplifier (Spectra-Physics TOPAS) to generate tunable pulses. This beam was used to resonantly excite the ground state of the LPB and generate a nonlinear response of the system. The two beams were cross-linearly polarized and were focused on a spot of ~3 μm. The residual pump signal was removed along the detection line by using a polarizer and a filter in momentum space.

### Calculation of polariton density

The polariton density has been calculated considering the visibility of the LPB at *k* = 0, which given the resonant excitation in reflection configuration, appears as a dip below the laser energy profile. A representative example of the dip visibility is shown in Supplementary Fig. 14 for the ML microcavity. We first considered the incident pulse energy, *E*_tot_ = *P/R*, where *P* is the incident average power and *R* the repetition rate (1 kHz), and then estimated the injected photon energy as follows:

$${E}_{{{\rm {INJ}}}}=\frac{{E}_{{{\rm {tot}}}}\int {A}_{{{\rm {abs}}},{{\rm {LP}}}}}{\int {A}_{{{\rm {laser}}},{{\rm {LP}}}}}$$

where $\int {A}_{{{\rm {abs}}},{{\rm {LP}}}}$ represents the integral of the dip of the LPB at *k* = 0, and $\int {A}_{{{\rm {laser}}},{{\rm {LP}}}}$ is the integral of the laser profile. Specifically, from the dip of the dispersion we estimate the injected number of photons (*n*_ph_) that can be related to the polariton density (*n*_pol_) through the photonic fraction (*C*) of the ground state, according to the following relation: ${n}_{{{\rm {ph}}}}={n}_{{{\rm {pol}}}}C$. The injected polariton density is then calculated as: ${n}_{{{\rm {pol}}}}=\frac{{n}_{{{\rm {ph}}}}}{C}=\frac{{E}_{{{\rm {inj}}}}}{C\,{A}_{{{\rm {beam}}}}{E}_{{{\rm {LP}}}}}$ where *E*_LP_ is the energy of the ground state and ${A}_{{{\rm {beam}}}}=\pi {D}^{2}/4$ is the beam area with a diameter, *D*, of ~3 μm, respectively. It is important to note that the presented calculation is a conservative approximation since we are considering that the total injected energy is completely converted into polaritons, thus neglecting scattering losses.

## Supplementary information

Supplementary Information

Description of Additional Supplementary Files

Supplementary Movie 1

Supplementary Movie 2

## Data Availability

All data needed to evaluate the conclusions in the paper are present in the paper and/or the Supplementary Materials. Additional data related to this paper may be requested from the authors.
