# Supplementary material for: Exciton polariton interactions in Van der Waals superlattices at room temperature
Source: Nat Commun. 2023 Mar 17;14:1512. doi: 10.1038/s41467-023-36912-3 (PMC10023709; doi:10.1038/s41467-023-36912-3)
Supplement: Supplementary file 1 — Supplementary Information [file 41467_2023_36912_MOESM1_ESM.pdf]

Exciton polariton interactions in Van der Waals superlattices at room temperature

Jiaxin Zhao[1, †], Antonio Fieramosca[1, †, *], Kevin Dini[1, †], Ruiqi Bao[1], Wei Du[1], Rui Su[1], Yuan Luo[2], Weijie Zhao[3,4], Daniele Sanvitto[5,6], Timothy C. H. Liew[1,7,*], Qihua Xiong[2,8,9,10,*]

Affiliations

[1]Division of Physics and Applied Physics, School of Physical and Mathematical Sciences, Nanyang Technological University, Singapore 637371

[2]State Key Laboratory of Low-Dimensional Quantum Physics and Department of Physics, Tsinghua University, Beijing 100084, P.R. China

[3]School of Physics, Frontiers Science Center for Mobile Information Communication and Security, Southeast University, Nanjing 211189, China.

[4]Purple Mountain Laboratories, Nanjing 211111, China.

[5]CNR NANOTEC Institute of Nanotechnology, via Monteroni, Lecce 73100, Italy

[6]INFN National Institute of Nuclear Physics, Lecce 73100, Italy

[7]MajuLab, International Joint Research Unit UMI 3654, CNRS, Université Côte d'Azur, Sorbonne Université, National University of Singapore, Nanyang Technological University, Singapore

[8]Frontier Science Center for Quantum Information, Beijing 100084, P.R. China

[9]Beijing Academy of Quantum Information Sciences, Beijing 100193, P.R. China

[10]Collaborative Innovation Center of Quantum Matter, Beijing 100084, P.R. China.

†These authors contributed equally to this work

*To whom correspondence should be addressed. Emails:
Qihua_xiong@tsinghua.edu.cn, tchliew@gmail.com and antonio.fieramosca@gmail.com

Supplementary Fig. 1: Optical characterization of samples with SiO_2 layer as spacer

Supplementary Fig. 2: Optical characterization of samples with hBN layer as spacer

Supplementary Fig. 3: The PL spectrum of monolayer, superlattice-N2, -N3 and -N4 before closing the microcavity

Supplementary Fig. 4: Optical characterization of the empty microcavity

Supplementary Fig. 5: Optical characterization of the microcavities with hBN spacer

Supplementary Fig. 6: Sketch of the optical setup for pump-probe experiments

Supplementary Fig. 7: Pump-probe experiment at time delay 0 for ML microcavity

Supplementary Fig. 8: Temporal dynamics of the energy renormalization for monolayer (device 1)

Supplementary Fig. 9: Temporal dynamics of the energy renormalization for monolayer (device 2)

Supplementary Fig. 10: The blueshift induced by the dark state population as a function of delay time, with four different exciton lifetimes

Supplementary Fig. 11. Comparison of the temporal dynamics of the superlattice-N3 for high (blue) pumping power, without any break in x-scale

Supplementary Fig. 12. Exciton-fraction controlled decay time for the superlattice-N3

Supplementary Fig. 13. The blueshift lifetime as a function of the disorder amplitude

Supplementary Fig. 14. Angle-resolved reflectivity map for the monolayer microcavity with the resonant pump

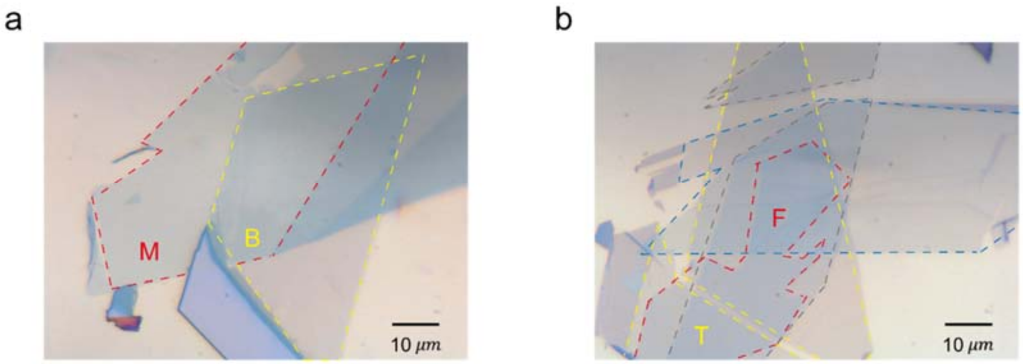

Supplementary Fig. 1. Optical characterization of samples with SiO_2 layer as spacer. (A) Optical image of monolayer (red dashed line and yellow dashed line) and superlattice N2 (the overlapped area) samples on bottom DBR. **(B)** Same as in a) but for superlattice N3 and N4 samples. The separated monolayers are indicated by red, yellow, blue, and gray dashed lines, respectively.

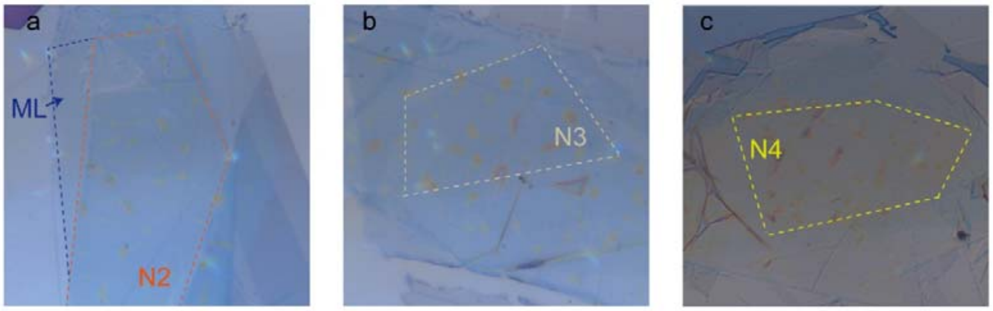

Supplementary Fig. 2. Optical characterization of sample with hBN layer as spacer. **(A)** Optical image of monolayer (blue dashed line) and superlattice-N2 (orange dashed line) samples on bottom DBR. Optical image of superlattice-N3 **(B)** and superlattice-N4 **(C)** samples on bottom DBR.

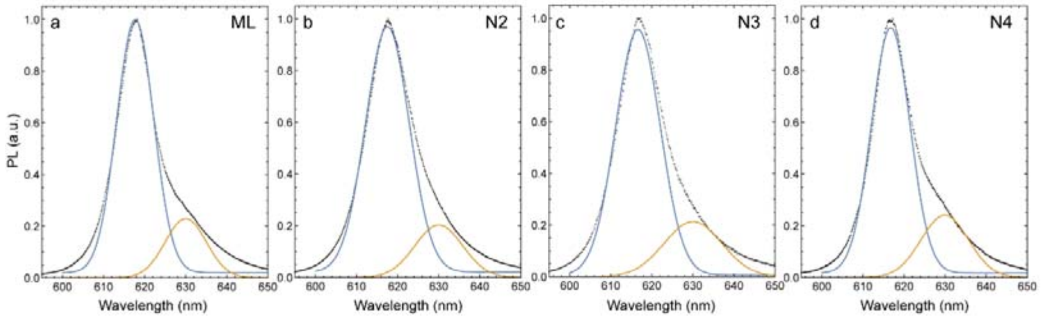

Supplementary Fig. 3. The PL spectrum (black line) of monolayer (A), superlattice-N2 (B), -N3 (C) and -N4 (D) at room temperature before closing the microcavity. The blue and orange lines, correspond to the A exciton and trion, respectively.

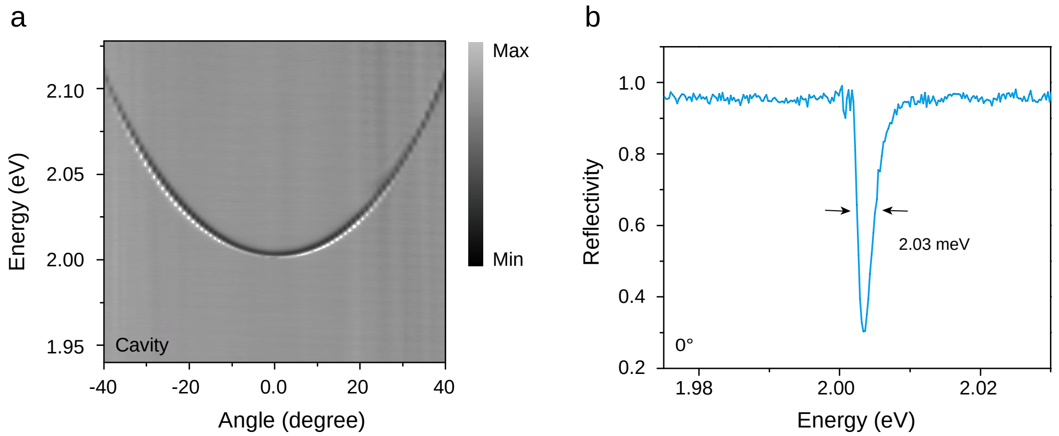

Supplementary Fig. 4. Optical characterization of the empty microcavity. (A) Angle-resolved reflectivity map of the bare microcavity mode. **(B)** The reflectivity spectrum of the cavity mode at normal incidence ($\theta = 0°$). The linewidth of the cavity mode is around 2 meV at 2.003 eV, corresponding to a quality factor of ~1000.

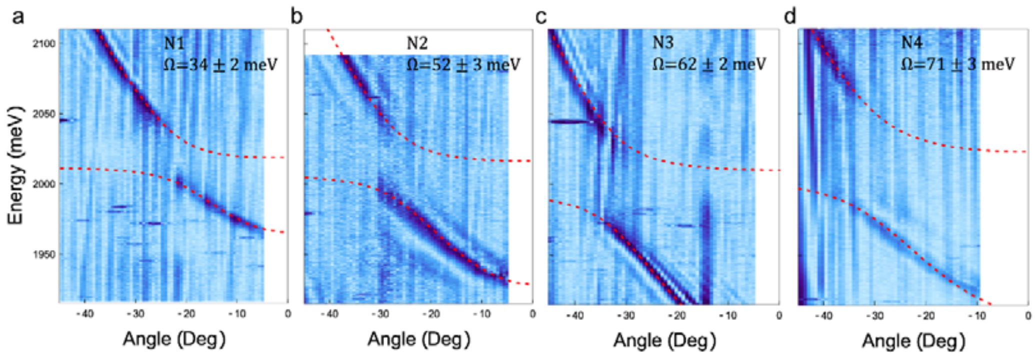

Supplementary Fig. 5. Optical characterization of the microcavities with hBN spacer. Angle-resolved reflectivity maps for the monolayer **(A)**, superlattice-N2 **(B)**, -N3 **(C)** and -N4 **(D)**.

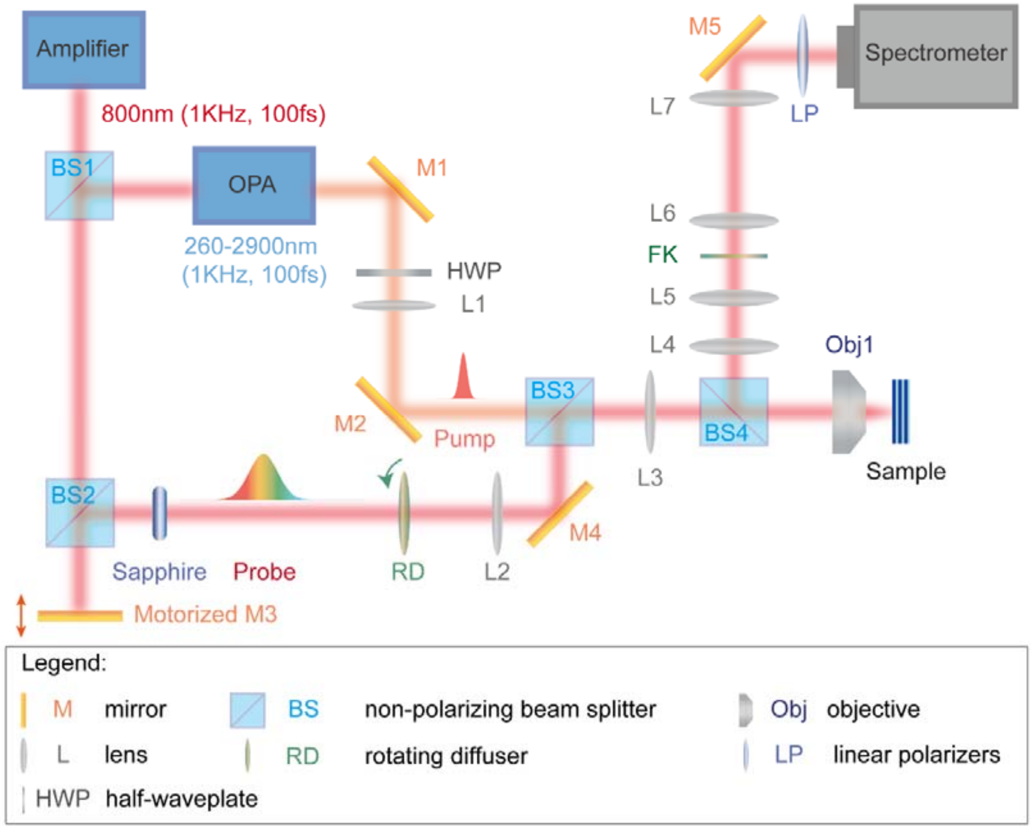

Supplementary Fig. 6. Sketch of the optical setup for pump-probe experiments. List of the optical components: M1, M2, M4 and M5 are mirrors; M3 is the mirror mounted in motorized linear stage to generate the time delay between pump and probe; BS1, BS2, BS3 and BS4 are non-polarizing beam splitters; FS is the notch filter used for pump pulses; FK is the filter in moment space to remove the pump beam; HWP is the half-waveplate and LP is linear polarizers, which used to further remove the pump beam; L1, L2, L3, L4, L5, L6 and L7 are convex lenses; Obj1 is the objective used for excitation and collection in reflection, with 100x magnification and 0.9 numerical aperture. RD is the rotating diffuser.

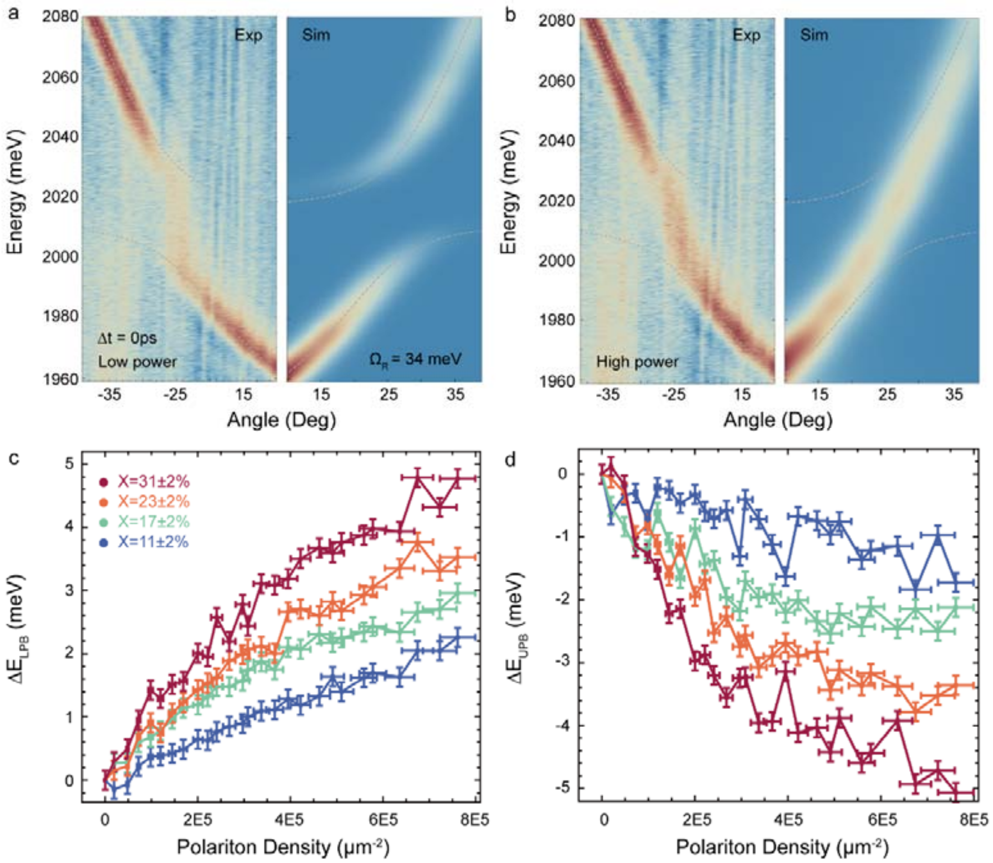

Supplementary Fig. 7. Pump-probe experiment at time delay 0 for ML microcavity. (A) Angle-resolved reflectivity map at high momenta for low (left) and high (right) excitation power. **(B)** Extracted energy shift as a function of the pumping power for LPB (blue points) and UPB (red points) for different excitonic fractions.

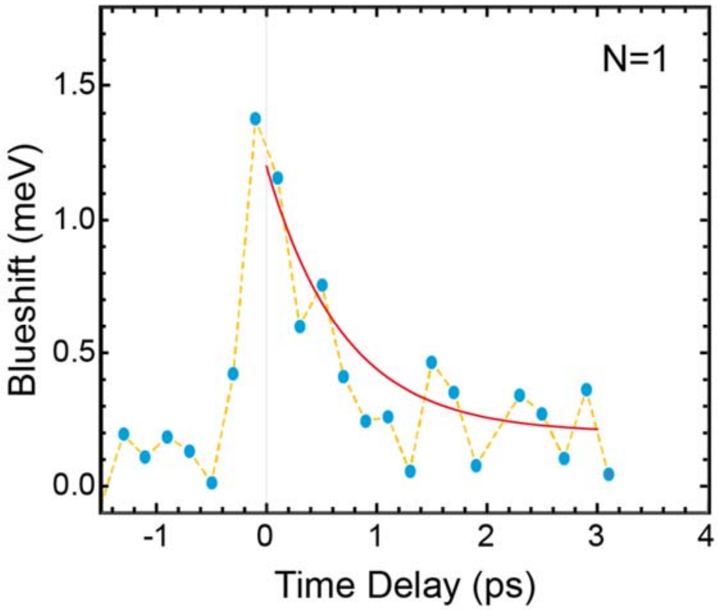

Supplementary Fig. 8. Temporal dynamics of the energy renormalization for monolayer (device 1). Evolution of LPB blueshift as a function of time delay *t* between pump and probe for monolayer microcavity.

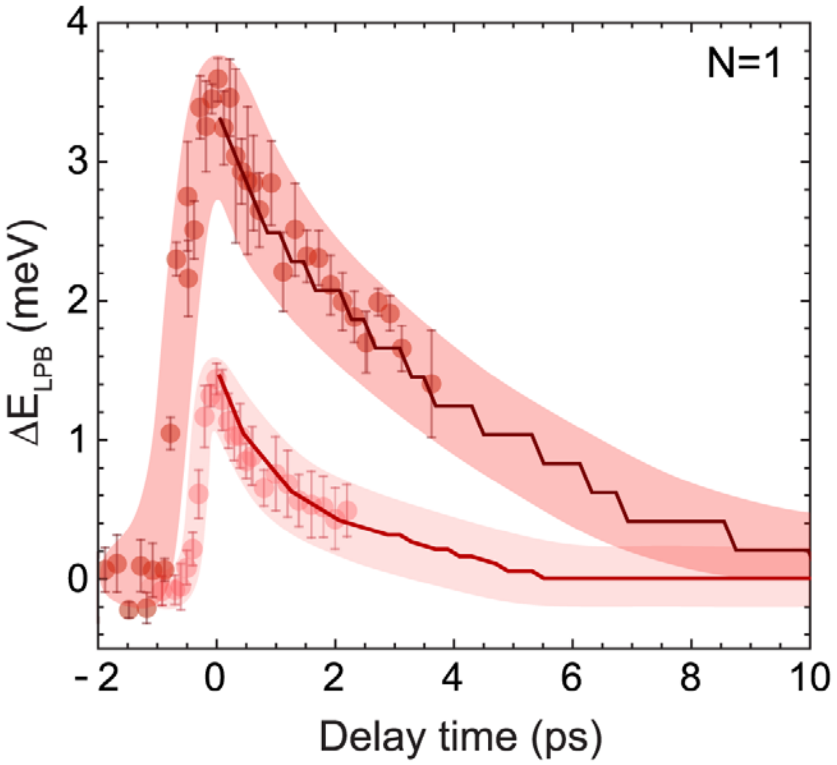

Supplementary Fig. 9. Temporal dynamics of the energy renormalization for monolayer (device 2). LPB blueshift as a function of the time delay between the pump and the probe for two different excitation powers, low (light red dots) and high (dark red dots, respectively). The solid lines represent the theoretical model.

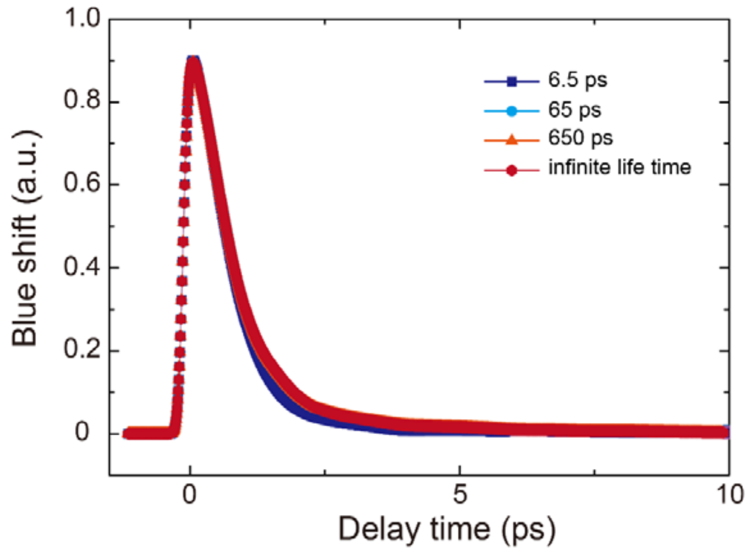

Supplementary Fig. 10. The blueshift induced by the dark state population as a function of delay time with four different exciton lifetimes. Temporal decay of the blueshift by taking into account different exciton lifetimes: 6.5 ps (dark blue), 65 ps (light blue), 650 ps (orange) and infinite (red), respectively.

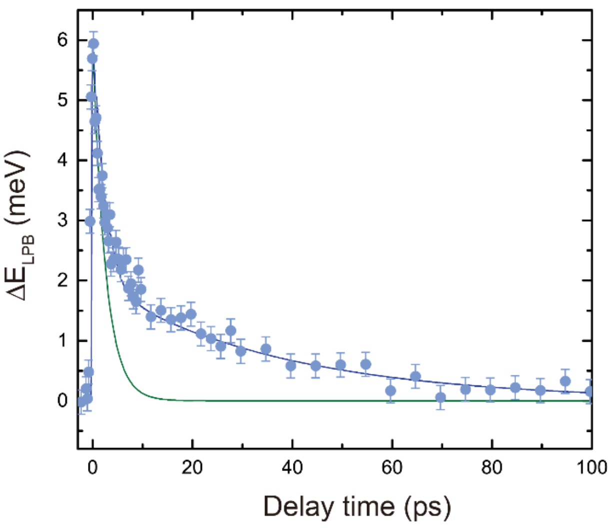

Supplementary Fig. 11. Comparison of the temporal dynamics of the superlattice-N3 for high (blue) pumping power, without any break in x-scale. The dots represent the experimental points about the blueshift as a function of the delay time for superlattice-N3. The solid blue line is the fit considering the model reported in the main text (*i.e.*, bi-exponential fit) for high pumping power, which well approximates the experimental points. The green line represents a single exponential decay which is capable to fit the experimental data within few picoseconds.

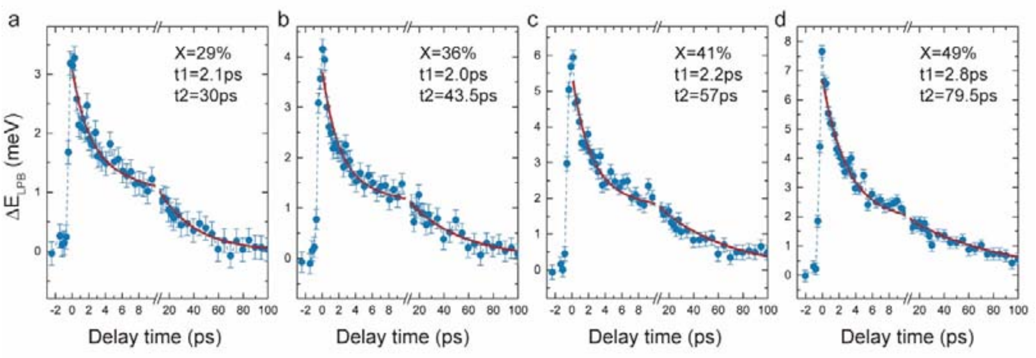

Supplementary Fig. 12. Exciton-fraction controlled decay time for the superlattice-N3. The energy decay of the LPB blueshift as a function of the time delay is shown in **(A)**, **(B)**, **(C)** and **(D)** for X=0.2, 0.3, 0.4 and 0.5, respectively.

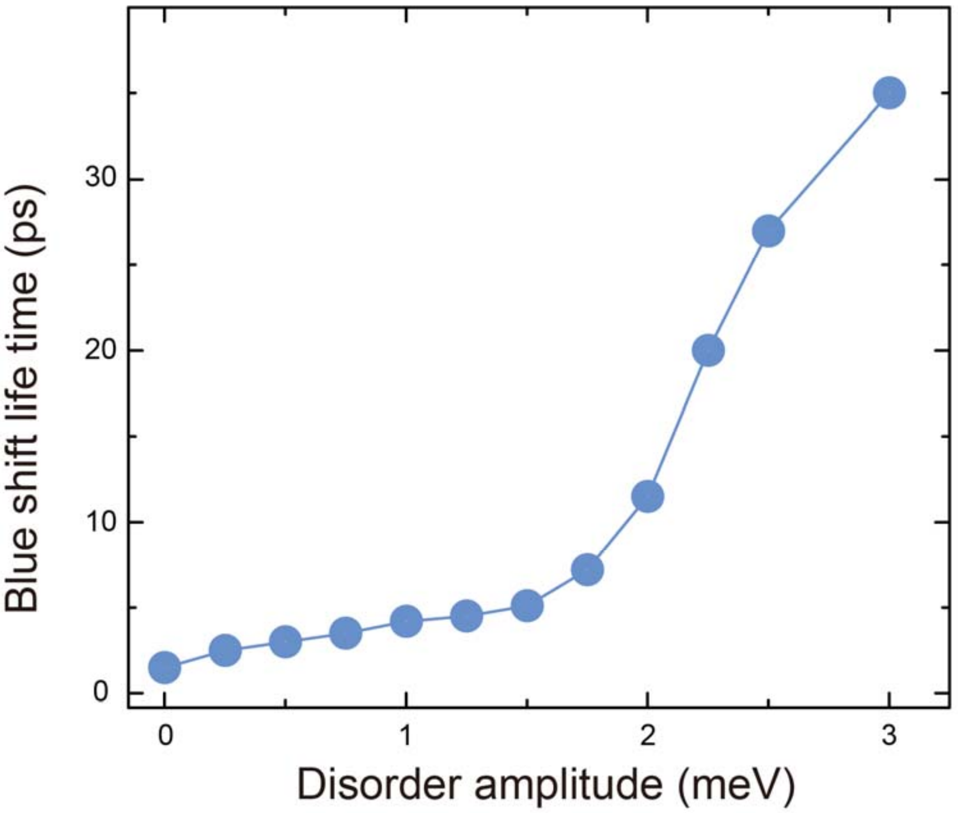

Supplementary Fig. 13. The blueshift lifetime as a function of the disorder amplitude. The bright excitons states need to scatter to obtain the phase mismatch and therefore populate the dark states. The counterpart is that to exit this dark state, the dark exciton needs to scatter back to the bright one. These scattering rates depend on the amplitude of the disorder and the population of each state which changes in time, i.e., is different at the moment of the pump and after 20 ps. Therefore, we simulated the dependency of the blueshift lifetime with respect to the amplitude of the random disorder.

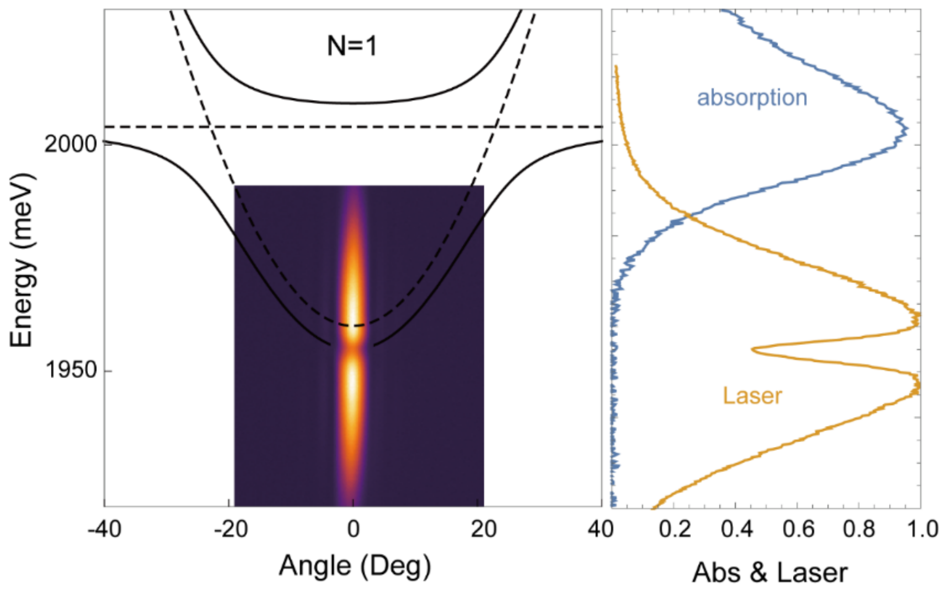

Supplementary Fig. 14. Angle-resolved reflectivity map for the monolayer microcavity with the resonant pump. Angle-resolved reflectivity map showing the pumping beam at k=0. The absorption of the TMD monolayer (blue line) and injected laser profile (orange line) are shown in the right panel. The dip visible below the laser profile represents the LPB mode at k=0.
