# Supplementary material for: Exciton polariton interactions in Van der Waals superlattices at room temperature
Source: Nat Commun. 2023 Mar 17;14:1512. doi: 10.1038/s41467-023-36912-3 (PMC10023709; doi:10.1038/s41467-023-36912-3)
Supplement: Supplementary file 2 — Description of Additional Supplementary Files [file 41467_2023_36912_MOESM2_ESM.pdf]

Title: Supplementary Movie 1

Description: Pump-probe experiment at time delay 0 for superlattice-N3 microcavity. Angle-resolved reflectivity map at high momenta with increasing of excitation power. The quenching of the Rabi splitting is highlighted

Title: Supplementary Movie 2

Description: Pump-probe experiment at time delay 0 for ML microcavity.
