# Supplementary figures and images for: Exciton polariton interactions in Van der Waals superlattices at room temperature
Source: Nat Commun. 2023 Mar 17;14:1512. doi: 10.1038/s41467-023-36912-3 (PMC10023709; doi:10.1038/s41467-023-36912-3)

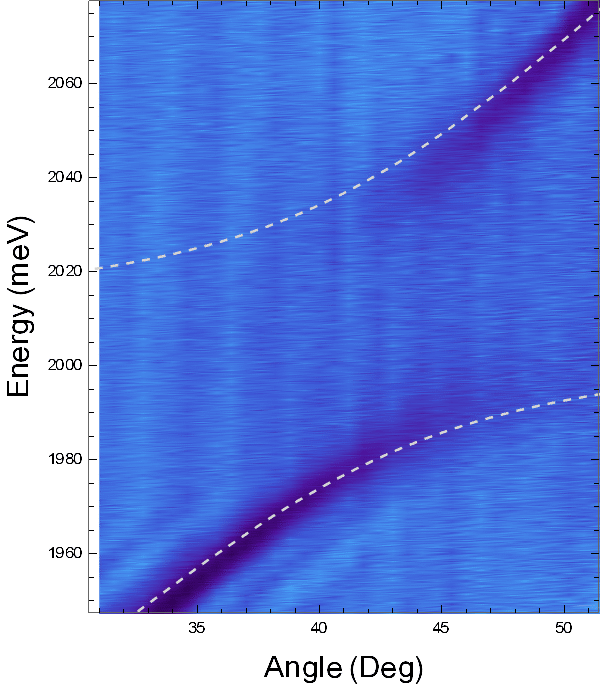

Supplement: Supplementary file 3 — Supplementary Movie 1 [file 41467_2023_36912_MOESM3_ESM.gif]

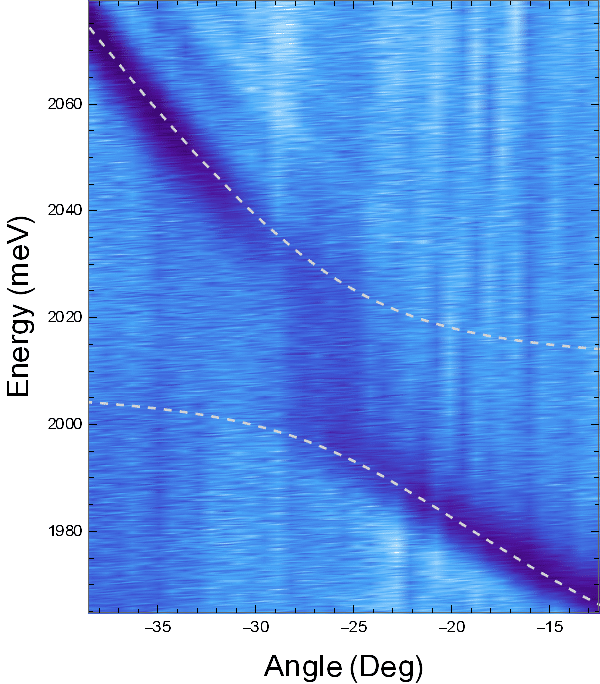

Supplement: Supplementary file 4 — Supplementary Movie 2 [file 41467_2023_36912_MOESM4_ESM.gif]
